# Understanding dynamics and overlapping epidemiologies of HIV, HSV-2, chlamydia, gonorrhea, and syphilis in sexual networks of men who have sex with men

**DOI:** 10.3389/fpubh.2024.1335693

**Published:** 2024-04-02

**Authors:** Ryosuke Omori, Hiam Chemaitelly, Laith J. Abu-Raddad

**Affiliations:** ^1^Division of Bioinformatics, International Institute for Zoonosis Control, Hokkaido University, Sapporo, Hokkaido, Japan; ^2^Infectious Disease Epidemiology Group, Weill Cornell Medicine-Qatar, Cornell University, Doha, Qatar; ^3^World Health Organization Collaborating Centre for Disease Epidemiology Analytics on HIV/AIDS, Sexually Transmitted Infections, and Viral Hepatitis, Weill Cornell Medicine-Qatar, Qatar Foundation - Education City, Cornell University, Doha, Qatar; ^4^Department of Population Health Sciences, Weill Cornell Medicine, Cornell University, New York, NY, United States; ^5^Department of Public Health, College of Health Sciences, QU Health, Qatar University, Doha, Qatar; ^6^College of Health and Life Sciences, Hamad Bin Khalifa University, Doha, Qatar

**Keywords:** HIV epidemiology, sexually transmitted infections/diseases, men who have sex with men, modeling, public health

## Abstract

**Introduction:**

We aimed to investigate the overlapping epidemiologies of human immunodeficiency virus (HIV), herpes simplex virus type 2 (HSV-2), chlamydia, gonorrhea, and syphilis in sexual networks of men who have sex with men (MSM), and to explore to what extent the epidemiology of one sexually transmitted infection (STI) relates to or differs from that of another STI.

**Methods:**

An individual-based Monte Carlo simulation model was employed to simulate the concurrent transmission of STIs within diverse sexual networks of MSM. The model simulated sexual partnering, birth, death, and STI transmission within each specific sexual network. The model parameters were chosen based on the current knowledge and understanding of the natural history, transmission, and epidemiology of each considered STI. Associations were measured using the Spearman’s rank correlation coefficient (SRCC) and maximal information coefficient (MIC).

**Results:**

A total of 500 sexual networks were simulated by varying the mean and variance of the number of partners for both short-term and all partnerships, degree correlation, and clustering coefficient. HSV-2 had the highest current infection prevalence across the simulations, followed by HIV, chlamydia, syphilis, and gonorrhea. Threshold and saturation effects emerged in the relationship between STIs across the simulated networks, and all STIs demonstrated moderate to strong associations. The strongest current infection prevalence association was between HIV and gonorrhea, with an SRCC of 0.84 (95% CI: 0.80–0.87) and an MIC of 0.81 (95% CI: 0.74–0.88). The weakest association was between HSV-2 and syphilis, with an SRCC of 0.54 (95% CI: 0.48–0.59) and an MIC of 0.57 (95% CI, 0.49–0.65). Gonorrhea exhibited the strongest associations with the other STIs while syphilis had the weakest associations. Across the simulated networks, proportions of the population with zero, one, two, three, four, and five concurrent STI infections were 48.6, 37.7, 11.1, 2.4, 0.3, and < 0.1%, respectively. For lifetime exposure to these infections, these proportions were 13.6, 21.0, 22.9, 24.3, 13.4, and 4.8%, respectively.

**Conclusion:**

STI epidemiologies demonstrate substantial overlap and associations, alongside nuanced differences that shape a unique pattern for each STI. Gonorrhea exhibits an “intermediate STI epidemiology,” reflected by the highest average correlation coefficient with other STIs.

## Introduction

Sexually transmitted infections (STIs) encompass a diverse group of pathogens, including viruses, bacteria, and parasites, and present a persistent global health challenge with ramifications for individuals, communities, and healthcare systems (1, 2). The intricate interplay of biological, behavioral, and social factors determines the transmission dynamics, prevalence, and the clinical, psychosocial, and economic burdens of STIs (1–3). Recognizing the impact of STIs on sexual and reproductive health, as well as the implications for the human immunodeficiency virus (HIV) epidemic, the World Health Organization (WHO) formulated the “Global Health Sector Strategy on STIs” with the aim to eliminate STIs as a public health concern (4, 5). The WHO has initiated efforts to enhance the understanding of STI epidemiology, estimate STI levels and trends, and build a case for investing in STI prevention, care, and the development of novel interventions, such as vaccines, to control infection transmission and mitigate the STI burden on individuals and societies (6–12).

STIs disproportionately affect specific populations, such as men who have sex with men (MSM) (13). The impact of various types and structures of sexual networks, whether among MSM or other populations, on infection transmission patterns and the epidemiology of STIs remains inadequately understood (14, 15). Given the shared mode of transmission within these networks, there is an inherent expectation that the epidemiology of different STIs would exhibit interconnections and overlaps (16–19).

However, different STIs possess distinct attributes that could shape their epidemiological patterns in divergent ways (15, 20). Specific STIs lead to lifelong infections that cannot be cured, such as HIV and herpes simplex virus type 2 (HSV-2), both of which are viral infections (15, 21, 22). In contrast, other STIs, including chlamydia, gonorrhea, and syphilis, three bacterial infections, can be naturally eliminated by the immune system after a relatively short period of infection or can be effectively cured (20). The likelihood of transmission per sexual act varies across different STIs and is influenced by the type of sexual activity, whether it is vaginal sex, anal sex, or oral sex (20–23). Given these differences in the natural history and transmissibility of STIs, distinct sexual risk behaviors and sexual-network statistics could influence the epidemiology of individual STIs in disparate ways (15).

We have developed an individual-based mathematical model to simulate the concurrent transmission of five major STIs—HIV, HSV-2, chlamydia, gonorrhea, and syphilis—within the sexual networks of MSM. The objective of this study is to investigate the epidemiological connections among these STIs and the ways in which their epidemiologies intersect. This was achieved by analyzing the epidemiological patterns of these STIs and their associations, estimating the prevalence of current infection, prevalence of ever infection, and the incidence rate of infection for each STI, as well as for concurrent infections.

While our understanding of the relationships between these STIs typically originates from individual-level associations identified in observational studies, this investigation delves into the population-level associations across these five STIs within diverse sexual networks that represent the global variation of MSM sexual networks. These networks vary in the mean and variance of the number of sexual partners for both short-term and long-term partnerships (15, 24), as well as in the extent of clustering (15, 25), concurrency (15, 26), and the tendency of individuals with similar numbers of partnerships to either connect or avoid each other (the latter referred to in the literature as degree correlation) (15, 25). As a result, this study establishes a theoretical foundation and framework to describe and comprehend the intertwined nature of the epidemiological links and overlapping patterns of these STIs.

## Methods

### Mathematical model

An individual-based Monte Carlo simulation model was developed to simulate the sexual networks of MSM and the concurrent transmission of HIV, HSV-2, chlamydia, gonorrhea, and syphilis within these networks. The foundation of this model is rooted in our previous work, specifically the model constructed for the transmission of HIV and HSV-2 infections (15). However, we expanded its scope to encompass the transmission of chlamydia, gonorrhea, and syphilis. This expanded modeling framework was developed with insights drawn from other individual-based STI models (20, 27).

The model simulated sexual partnering, birth, death, and STI transmission within each specific sexual network. In each simulation run, each individual underwent events such as birth, death, formation or dissolution of long-term or short-term partnerships, or acquisition of an STI, with event-specific probabilities at each time step. The transmission of STIs was assumed to occur exclusively through anal sex, considering that this mode of transmission is the primary route of transmission among MSM and due to the availability of data on STI transmission through anal sex.

The probability of STI transmission within a sexual partnership was calculated using the binomial model (28, 29), which considered the probability of STI transmission per sexual act and the total number of acts within the partnership. An initial population of 2,000 individuals was used in the simulation. Individuals who left the network due to death were replaced by newborns without any STI history who age and reenter the sexual network, thus maintaining the cohort size of the simulated sexually active population.

The model included individuals of all age groups, but only those within the 15–64 years age range were considered sexually active and at risk of STI acquisition. Individuals older than 64 years of age, even if sexually active, do not appear to play a critical role in STI transmission in the population, which tends to be focused among young populations (30, 31). The progression through the natural history stages of each STI was stochastically simulated using an exponential distribution, with the rate determined by the inverse of the duration of each stage. The coding of the model and conduct of simulations were executed using the C programming language. The statistical analyses were conducted in R 4.1.2.

### Infection natural history and transmission

The model parameters were chosen based on the current knowledge and understanding of the natural history, transmission, and epidemiology of each considered STI (Supplementary Table S1). These parameters characterize the attributes of each STI, including the structure of infection stages, stage durations, shedding frequency, transmission probability per sexual act categorized by infection stage, proportion of symptomatic versus asymptomatic infections, proportion of infections effectively treated, proportion of infections conferring immunity post-treatment or through natural clearance, and duration of immunity.

Treated individuals without immunity were presumed susceptible to infection, and they were considered non-infectious once treatment is initiated. HIV antiretroviral therapy and HSV-2 antiviral therapy were not included in the model to focus on simulating the natural dynamics of HIV and HSV-2 epidemics. An additional consideration is achieving a manageable complexity of the model, given that the current model is already quite complex by incorporating the concurrent transmission of five different STIs.

Primary sources of model parameters are our previous work concerning HIV and HSV-2 infections (15, 21, 22, 32, 33), and a comprehensive investigation into STI models and their parameterization for chlamydia, gonorrhea, and syphilis conducted by Johnson and Geffen (20). In instances where data on the transmission probability per one anal sex act were unavailable, but the relative infectivity of anal compared to vaginal sex was known, this probability was calculated by multiplying the transmission probability per coital act for vaginal sex by this relative infectivity (Supplementary Table S1).

Due to the conflicting evidence regarding the biological interaction between HIV and other STIs (including HSV-2) (19, 34, 35), no biological HIV/STI interaction was assumed. A summary of used parameters and their respective references is presented in Supplementary Table S1. Additional information for each specific STI can be found in earlier publications (15, 20–22, 28, 32, 33).

### Sexual behavior, sexual networks, and network statistics

The model simulated two types of sexual partnerships: long-term (spousal) partnerships and short-term (casual) partnerships. Long-term partnerships were assumed to form and dissolve at a specific rate with no polygamy, lasting for an average duration of 5 years. Short-term partnerships were envisaged to form and dissolve among individuals in long-term partnerships and those not engaged in such partnerships, but at distinct rates, and with an average duration of 2 weeks. The number of short-term partnerships per year followed a gamma distribution (24). Both the count of partnerships and the frequency of sexual acts were assumed to be dependent on age, peaking between 20 and 30 years of age, and gradually declining with increasing age. These patterns were based on empirical self-reported data on sexual behavior (36, 37).

A weighted random network is a network in which each individual randomly selects partners from the entire population, but each individual may have a different propensity to form a partnership, a phenomenon known as proportionate mixing (38). In such a network, the degree correlation and clustering coefficient are determined by the degree (number of partners) distribution (15, 24). Degree correlation measures the correlation between the number of partners of each partner in a partnership (15, 25). The clustering coefficient measures the extent to which individuals in the network tend to form clusters (15, 25).

To optimize the diversity of simulated sexual networks and ensure their representation of real-world MSM sexual networks on a global scale (39–43), degree correlation and clustering coefficient were deliberately varied independently. This was achieved through a “rewiring” methodology, employing a technique previously developed (44). This technique allowed for the independent variation of degree correlation and clustering coefficient while maintaining the same degree distribution (15, 44, 45), and it involved the use of a tuning factor with two parameters, denoted as κcorr and κclus, which individually governed the degree correlation and clustering coefficient, respectively (15, 44, 45). In the scenario where κcorr = κclus = 0, the network transforms into a weighted random network.

Consequently, the simulated networks exhibited varying network statistics, including the population’s mean and variance of the number of partners within the past year for both long-term and short-term partnerships, the degree correlation for both short-term and all partnerships, the clustering coefficient for short-term partnerships, and the prevalence of concurrency for short-term and all partnerships (15). Concurrency prevalence measures the proportion of individuals who are engaged with two or more partners at a given time (15, 26). Further description of these network statistics in simulated MSM sexual networks has been previously reported (15).

### Model simulations and outcome measures

A total of 500 sexual networks were simulated by varying the mean and variance of the number of partners for both short-term and all partnerships, degree correlation (via κcorr), and clustering coefficient (via κclus). These network parameters were sampled from plausible ranges (Supplementary Table S1) to represent the diverse landscape of sexual networks among MSM.

For each network, STI transmission was also simulated. The initial setup for each simulation involved seeding 10 infected individuals for each STI, followed by a “burn-in” period of 200 years to attain equilibrium. The decision to use 10 seeds, rather than just one, was made to decrease the likelihood of stochastic extinction for any of the considered STIs. The long burn-in period of 200 years was utilized to establish endemic equilibrium. All analyses were conducted at this endemic equilibrium to isolate the epidemiological effects from transient temporal variations. Prevalence levels at equilibrium are also unaffected by the initial number of seeds. Additionally, to counter stochastic extinction within the finite simulated network, an average of one STI infection for each of the simulated STIs was introduced annually. These introductions occurred randomly while maintaining the average of one infection introduced per year.

Consequently, we generated 500 STI epidemics, each within a distinct sexual network. The means of the distributions of outcome measures across the 500 simulation runs were employed for generating model predictions. The selection of this simulation count strikes a balance between computational efficiency and the requirement for a broad spectrum of networks and STI epidemics, all while obtaining outcome measures with adequately narrow confidence intervals to support valid inferences. The utilization of 500 simulations was determined to yield minimal fluctuation in the distribution of the outcome measures.

Prevalence of current infection, prevalence of ever infection, and incidence rate of infection for each STI were calculated in each simulation run. The prevalence of current infection was defined as the proportion of the population currently infected with each STI. The prevalence of ever infection was defined as the proportion of the population ever infected with each STI. The incidence rate for each STI was defined as the number of new infections per 100 person-years.

To investigate the associations between STI outcome measures, two distinct methods were employed: Spearman’s rank correlation coefficient (SRCC) and maximal information coefficient (MIC). These methods collectively offer a nuanced description of the association between pairs of STIs. SRCC serves as a measure of the correlation between the rankings of two variables. In essence, it gauges the extent to which the relationship between the two variables can be characterized by a monotonic function. On the other hand, the MIC measures the quantitative strength of the association between two variables, encompassing both linear and non-linear relationships. Both SRCC and MIC operate without making any assumptions regarding the distribution of the variables. The resulting values from both metrics range between 0 (indicating no association) and 1 (indicating a perfect association).

Bootstrap resampling, employing 1,000 iterations, was used to derive estimates for the 95% confidence intervals (CI) of both SRCC and MIC. Within this resampling framework, the median was utilized as the point estimate. Evaluating the strength of associations hinged on predefined thresholds: associations were deemed strong if SRCC or MIC ≥0.6, of intermediate strength if 0.2 ≤ SRCC or MIC <0.6, and weak if SRCC or MIC <0.2.

### Oversight

This is a mathematical modeling study and did not involve research with human subjects; therefore, no ethical approval or consent procedures were required.

## Results

### Prevalence and incidence of STIs

Table 1 presents the mean prevalence of current infection, mean prevalence of ever infection, and mean incidence rate for the five STIs across the 500 simulated STI epidemics in the 500 distinct MSM sexual networks.

**Table 1:** Mean prevalence of current infection, mean prevalence of ever infection, and mean incidence rate of infection for the five STIs across the 500 simulated STI epidemics in the 500 diverse MSM sexual networks.

Epidemiological measure	HIV	HSV-2	Chlamydia	Gonorrhea	Syphilis
Prevalence of current infection (in %)	11.8	41.1	7.9	2.7	4.5
Prevalence of ever infection (in %)	11.8	41.1	84.1	58.9	21.2
Incidence rate of infection (per 100 person-years)	1.3	2.7	14.0	14.1	1.9

HIV denotes human immunodeficiency virus; HSV-2, herpes simplex virus type 2; MSM, men who have sex with men; STI, sexually transmitted infection.

The STI with the highest prevalence of current infection was HSV-2 at 41.1%, followed by HIV at 11.8%, chlamydia at 7.9%, syphilis at 4.5%, and gonorrhea at 2.7% (Table 1). For the prevalence of ever infection, chlamydia had the highest prevalence at 84.1%, followed by gonorrhea at 58.9%, HSV-2 at 41.1%, syphilis at 21.2%, and HIV at 11.8%. Gonorrhea had the highest incidence rate of infection at 14.1 per 100 person-years, followed by chlamydia at 14.0, HSV-2 at 2.7, syphilis at 1.9, and HIV at 1.3 per 100 person-years.

### Relationship between the prevalences of STIs

**Figure 1:**
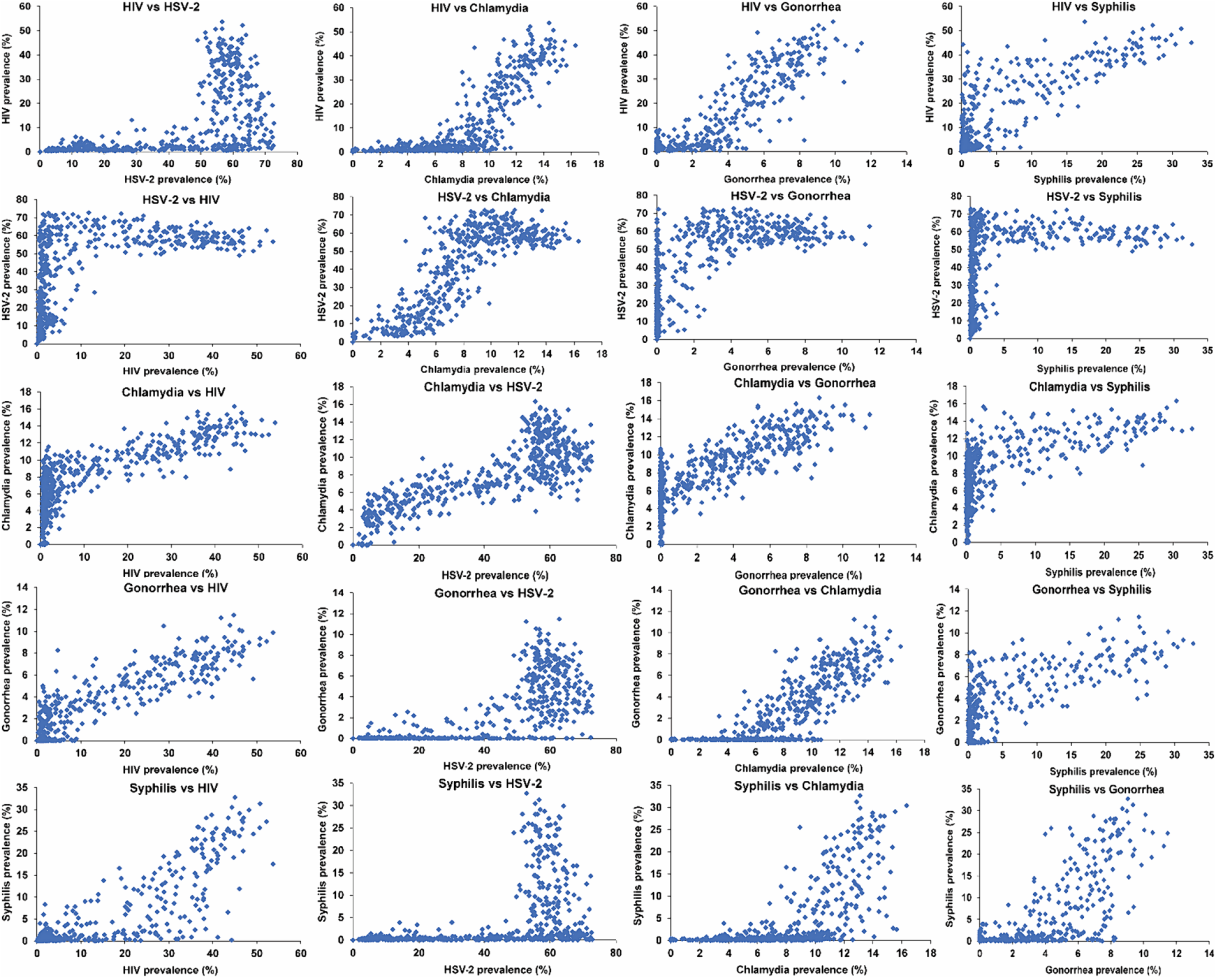
Scatterplots depicting the relationship between the prevalences of current infection for the five STIs across the 500 simulated STI epidemics in the 500 diverse MSM sexual networks. HIV denotes human immunodeficiency virus; HSV-2, herpes simplex virus type 2; MSM, men who have sex with men; STI, sexually transmitted infection.

Figure 1 illustrates scatterplots depicting the relationship between the prevalences of current infection for the different STIs across the 500 simulations. A large threshold effect emerged in relation to the prevalence of HIV, gonorrhea, and syphilis, with respect to the prevalence of HSV-2. Specifically, low or vanishing prevalences of HIV, gonorrhea, and syphilis were evident within networks where HSV-2 prevalence remained below 40%. However, at higher HSV-2 prevalence levels, the prevalences of HIV, gonorrhea, and syphilis exhibited a rapid increase, even with a slight rise in HSV-2 prevalence. Conversely, the prevalence of chlamydia demonstrated a monotonic increase with HSV-2 prevalence, without an apparent threshold effect.

A similarly large threshold effect was observed for the prevalence of HIV, gonorrhea, and syphilis with respect to the prevalence of chlamydia. Above a distinct threshold of chlamydia prevalence, varying for each STI, the prevalences of HIV, gonorrhea, and syphilis exhibited a rapid increase, even with a minor rise in chlamydia prevalence.

A small threshold effect was observed for the prevalence of HIV and syphilis in relation to the prevalence of gonorrhea. A smaller threshold effect was observed for the prevalence of syphilis in relation to the prevalence of HIV. However, no threshold effect was observed for the prevalence of any of the STIs in relation to syphilis prevalence. The prevalences of all STIs generally increased with increasing syphilis prevalence, except for HSV-2, which remained relatively constant with increasing syphilis prevalence, reaching saturation at low syphilis prevalence.

### Measures of association between the different STIs

Table 2 displays the SRCC and MIC values for the associations between the prevalences of current infection for each pair of STIs. In general, both SRCC and MIC demonstrated comparable values for each STI pair, revealing moderate to strong associations. While no association was weak, the strength of the associations did vary across the different STI pairs.

**Table 2:** Correlations between current infection prevalences for each pair of STIs, along with their 95% confidence intervals, estimated using (A) Spearman’s rank correlation coefficient and (B) the maximal information coefficient.

(A) Spearman’s rank correlation coefficient (SRCC)						Average
	HIV	HSV-2	Chlamydia	Gonorrhea	Syphilis	SRCC
HIV		0.65 (0.60, 0.70)	0.82 (0.78, 0.85)	0.84 (0.80, 0.87)	0.73 (0.68, 0.78)	0.76
HSV-2	0.65 (0.60, 0.70)		0.77 (0.72, 0.81)	0.70 (0.65, 0.74)	0.54 (0.48, 0.59)	0.67
Chlamydia	0.82 (0.78, 0.85)	0.77 (0.72, 0.81)		0.82 (0.78, 0.84)	0.70 (0.64, 0.74)	0.78
Gonorrhea	0.84 (0.80, 0.87)	0.70 (0.65, 0.74)	0.82 (0.78, 0.84)		0.70 (0.64, 0.75)	0.77
Syphilis	0.73 (0.68, 0.78)	0.54 (0.48, 0.59)	0.70 (0.64, 0.74)	0.70 (0.64, 0.75)		0.67
(B) Maximal information coefficient (MIC)						
	HIV	HSV-2	Chlamydia	Gonorrhea	Syphilis	MIC
HIV		0.67 (0.58, 0.76)	0.73 (0.67, 0.81)	0.81 (0.74, 0.88)	0.64 (0.57, 0.71)	0.71
HSV-2	0.67 (0.58, 0.76)		0.77 (0.70, 0.84)	0.78 (0.71, 0.86)	0.57 (0.49, 0.65)	0.70
Chlamydia	0.73 (0.67, 0.81)	0.77 (0.70, 0.84)		0.77 (0.70, 0.83)	0.58 (0.52, 0.65)	0.71
Gonorrhea	0.81 (0.74, 0.88)	0.78 (0.71, 0.86)	0.77 (0.70, 0.83)		0.62 (0.55, 0.69)	0.75
Syphilis	0.64 (0.57, 0.71)	0.57 (0.49, 0.65)	0.58 (0.52, 0.65)	0.62 (0.55, 0.69)		0.60

The correlation coefficients were computed across the 500 simulated STI epidemics in the 500 diverse MSM sexual networks.

HIV denotes human immunodeficiency virus; HSV-2, herpes simplex virus type 2; MSM, men who have sex with men; STI, sexually transmitted infection.

The strongest association was observed between HIV and gonorrhea, yielding an SRCC of 0.84 (95% CI: 0.80–0.87) and an MIC of 0.81 (95% CI: 0.74–0.88). This heightened strength stems from the similarity in the sustainability threshold for infection transmission (15), along with a similar dependence on the overall level of sexual risk behavior beyond this threshold. This can be indirectly inferred from Figure 1 for the relationship with HSV-2 prevalence, noting that HSV-2 prevalence proxies the network’s overall level of sexual risk behavior. A closely strong association was also identified between HSV-2 and chlamydia, revealing an SRCC of 0.77 (95% CI: 0.72–0.81) and an MIC of 0.77 (95% CI: 0.70–0.84).

The weakest association was found between HSV-2 and syphilis, resulting in an SRCC of 0.54 (95% CI: 0.48–0.59) and an MIC of 0.57 (95% CI: 0.49–0.65). This outcome is due to the very different thresholds of sustainability for both infections as well as the saturation of HSV-2 prevalence at low syphilis prevalence. Similarly, the association between syphilis and chlamydia was relatively weak with an SRCC of 0.70 (95% CI: 0.64–0.74) and an MIC of 0.58 (95% CI: 0.52–0.65).

Gonorrhea demonstrated the strongest associations with the other STIs, as evident from the SRCC and MIC values for each association, as well as across all STIs (Table 2). Conversely, syphilis exhibited the weakest associations with the other STIs, primarily due to its higher threshold for sustained transmission.

Supplementary Figure S1 presents scatterplots illustrating the relationship between the prevalences of ever infection for the different STIs across the 500 simulations, while Supplementary Table S2 provides the SRCC and MIC values for the associations. Notably, in sexual networks where the prevalence of HIV or syphilis reached a few percentage points, nearly the entire population had been infected at least once over their lifetime by both chlamydia and gonorrhea.

Supplementary Figure S2 displays scatterplots illustrating the relationship between the incidence rates of infection for the different STIs across the 500 simulations, while Supplementary Table S3 provides the SRCC and MIC values for these associations. Notably, a strong linear relationship is observed between the incidence rates of chlamydia and gonorrhea.

### Distribution of overlap of current and ever STI infections

**Figure 2:**
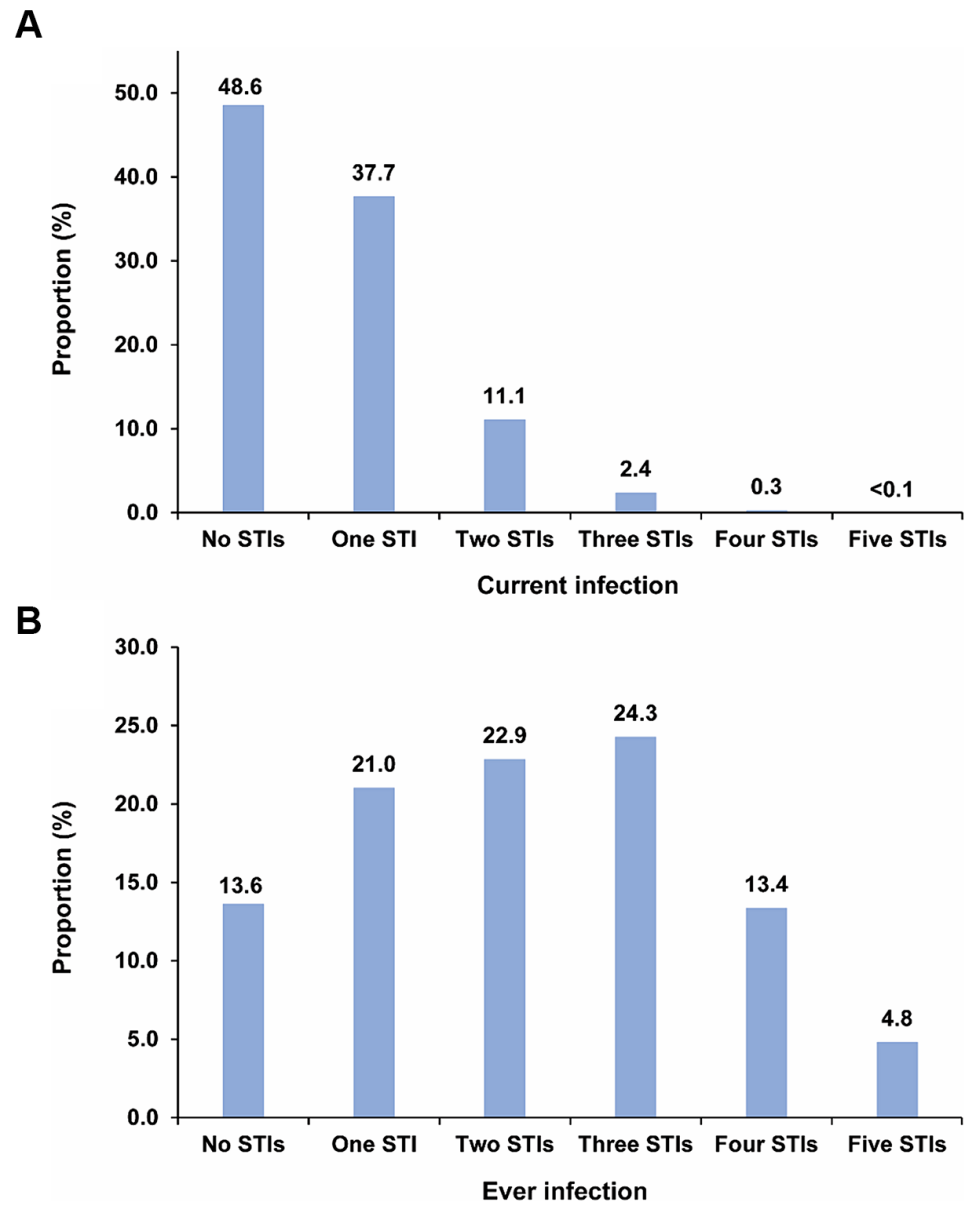
Distribution of **(A)** mean proportion of the population with no, one, two, three, four, and five concurrent STI infections, and **(B)** mean proportion of the population with no, one, two, three, four, and five ever STI infections across the 500 simulated STI epidemics in the 500 diverse MSM sexual networks. HIV denotes human immunodeficiency virus; HSV-2, herpes simplex virus type 2; MSM, men who have sex with men; STI, sexually transmitted infection.

Figure 2A illustrates the mean proportion of the population with no, one, two, three, four, and five concurrent STI infections across the 500 simulated STI epidemics. Nearly half of the population (48.6%) had no current STI infection at any given time. Meanwhile, the proportions of the population with one, two, three, four, and five concurrent STI infections were 37.7, 11.1, 2.4, 0.3, and < 0.1%, respectively. Supplementary Table S4 explicitly provides the proportions for each specific combination of the five considered STIs, thereby highlighting the combinations that contributed most to these proportions. HSV-2 was distinctly the most common current STI infection across all STI combinations.

Figure 2B displays the mean proportion of the population with no, one, two, three, four, and five ever STI infections across the 500 simulations. Only 13.6% of the population had never experienced any STI infection. In contrast, the proportions of the population with one, two, three, four, and five ever STI infections were 21.0, 22.9, 24.3, 13.4, and 4.8%, respectively. Supplementary Table S5 explicitly provides the proportions for each specific combination of the five considered STIs, thereby highlighting the combinations that contributed most to these proportions. Chlamydia followed by gonorrhea were the most common ever STI infections among all STI combinations.

## Discussion

Our study investigated the transmission dynamics and interconnected epidemiologies of HIV, HSV-2, chlamydia, gonorrhea, and syphilis across a broad spectrum of MSM sexual networks that aim to emulate real-world MSM sexual networks on a global scale (39–43). The epidemiological patterns of these STIs exhibited substantial overlap and generally strong associations, a consequence of their shared mode of transmission and propagation within the same sexual networks. These findings vividly illustrate substantial intersections in both present and ever exposure to different STIs, with a significant proportion of the population carrying current STI infections at any given time or having experienced infection with one or more STIs over their lifetime.

However, the findings also reveal clear and nuanced distinctions among the epidemiologies of these STIs, stemming from variations in their natural histories, levels of infectiousness, and divergent impacts of various sexual-network statistics on STI transmission, including mean and variance in the number of partners, degree correlation, clustering, and concurrency, as previously suggested (15).

The results show that these differences give rise to a threshold effect in the relationship between STIs. This effect emerges because certain sexual networks can be conducive to sustaining the transmission of specific STIs while not favoring the transmission of others. HSV-2 and chlamydia are the most easily propagating STIs. Many networks possess a sufficient level of sexual networking to sustain the transmission of these two infections but lack the necessary conditions for the sustained transmission of HIV, gonorrhea, and syphilis. Syphilis is the least sustainable STI, demanding a high level of risk behavior in the network to maintain its transmission. Consequently, networks where syphilis transmission is sustainable encompass high levels of risk behavior, enabling the transmission of all the other STIs, as indicated earlier (13). In such networks, most of the population would have been infected by HSV-2, and nearly all of the population would have been infected at least once with chlamydia and gonorrhea.

The association and overlapping epidemiologies between any pair of STIs are strongly affected by the presence of this threshold effect. Another critical factor is how rapidly the prevalence of one STI increases in relation to the prevalence of the other STI when both STIs are above their sustainability threshold. The third factor is a saturation effect, particularly noteworthy when one STI has a much lower sustainability threshold than the other STI. An illustrative example is the relationship between HSV-2 and syphilis. While HSV-2 can be easily sustained in sexual networks, syphilis necessitates a high level of sexual networking to maintain transmission—a threshold at which HSV-2 prevalence would have already reached its peak level of prevalence, that is at a point of saturation.

These three factors collectively determine the degree of overlap and similarity in the epidemiology of one STI relative to another. The epidemiology of gonorrhea can be described as “intermediate STI epidemiology,” as this infection requires an intermediate level of sexual networking for sustainability compared to other STIs, and its prevalence exhibits the highest average correlation coefficient with the other STIs. Similarly, HIV epidemiology demonstrates a close link to gonorrhea epidemiology. In contrast, the dynamics of HSV-2 and chlamydia versus syphilis highlight the spectrum’s limits among these STIs, representing the most easily and most challenging to propagate among them in sexual networks.

The results highlight distinct hierarchies in terms of STI current infection prevalence, ever infection prevalence, and incidence rate. The highest prevalence of current infection is observed for HSV-2, followed by HIV, chlamydia, gonorrhea, and syphilis. The highest prevalence of ever infection is seen for chlamydia, followed by gonorrhea, HSV-2, syphilis, and HIV. As for the incidence rate, gonorrhea leads, followed by chlamydia, syphilis, HSV-2, and HIV. These varying hierarchies are a manifestation of specific factors that differ across STIs, including infection duration, natural and treatment curability, duration of immunity against reinfection, and transmission probability per sexual act, as well as differential effects of sexual-network statistics.

These findings hold implications for public health responses to these STIs. The threshold effect underscores the importance of understanding how the impact of interventions against a specific STI varies depending on its proximity to the threshold. Largest impact and cost-effectiveness are likely to occur when the infection is just above its threshold, whereas the least impact and cost-effectiveness are observed when the infection is at the extremes, either well below or well above this threshold. Moreover, the strong associations between STIs suggest that interventions targeting one specific STI have implications for others as well, necessitating a comprehensive approach that consider these implications. This also highlights the potential for synergies by tailoring interventions to address multiple STIs simultaneously.

This study has limitations. Firstly, the predictions were based on the current understanding of the natural history of the five STIs. However, the natural histories of chlamydia, gonorrhea, and syphilis are still inadequately understood, including the effects of prior infections on subsequent ones. Secondly, the model simulated STI transmission only through anal sex acts, even though some of these STIs can be transmitted through other sexual and non-sexual modes. Thirdly, the study did not simulate transmission links with non-male partners or the broader population beyond MSM, but this is likely to have a less significant role in the transmission dynamics among MSM. Fourthly, aside from standard of care treatments for chlamydia, gonorrhea, and syphilis, no other interventions were included or simulated. Future studies may explore the incorporation of other interventions, such as HIV antiretroviral therapy, pre-exposure prophylaxis, and doxycycline post-exposure prophylaxis, to assess whether they affect the conclusions drawn in the present study and to investigate their impact on STI transmission within sexual networks, as well as their effectiveness. HIV antiretroviral therapy, in particular, may have significant consequences as it affects not only HIV transmission but also disease mortality, which varies based on sexual risk behavior. These variations indirectly influence the dynamics of the other STIs. Fifthly, although the concurrent transmission of the five STIs was simulated, the patterns observed might differ if HIV infection was not included, given the differential effects of AIDS mortality on individuals with different sexual behavior levels. Sixthly, testing practices were not explicitly modeled; instead, their effect was incorporated by assigning a probability for an infection to be treated, as informed by data (Supplementary Table S1). Future studies may wish to explore various testing practices and their implications for STI transmission patterns. Lastly, we assumed no biological interactions between HIV and other STIs, despite observational evidence suggesting the potential for such interactions (19, 34, 35).

Despite these limitations, this study provides critical and far-reaching analytical insights into the dynamics of STI transmission among diverse MSM sexual networks. Moreover, the study uniquely investigates the population-level associations between these STIs, which is a challenging and rarely explored area due to the requirement for data on distinct populations and sexual networks, in contrast to the individual-level associations that are more easily examined in conventional epidemiologic studies. The approach presented in this study is adaptable and can be extended to explore various other research questions in future studies. For instance, it can be utilized to investigate associations by age, assess the overlap of STIs among different age groups, evaluate the impact of a range of interventions targeting each of these STIs, and investigate the associations within heterosexual sex networks. Such extensions would provide insights into the dynamics of STI transmission and contribute to the development of effective intervention strategies.

## Conclusion

In conclusion, the epidemiological dynamics of HIV, HSV-2, chlamydia, gonorrhea, and syphilis reveal a significant degree of overlap and substantial associations, driven by their shared mode of transmission within the same sexual networks. Nevertheless, beneath these commonalities, intricate differences contribute to distinctive epidemiologies for these STIs. These variations emerge from the interplay of threshold and saturation effects, which vary across STIs, alongside differing dependencies of one STI’s prevalence on another’s. Furthermore, diverse hierarchies in terms of current infection prevalence, ever infection prevalence, and incidence rate exist among these STIs, reflecting their distinct attributes such as infection duration, natural and treatment curability, duration of immunity, and infectiousness. Notably, gonorrhea exhibits an “intermediate STI epidemiology,” characterized by the highest average correlation coefficient with other STIs. In contrast, the epidemiologies of HSV-2 and chlamydia versus syphilis delineate the spectrum’s boundaries among these STIs.

## Data availability statement

The original contributions presented in the study are included in the article/Supplementary material, further inquiries can be directed to the corresponding authors.

## Author contributions

RO: Formal analysis, Investigation, Methodology, Validation, Writing – review & editing, Writing – original draft. HC: Formal analysis, Writing – original draft, Writing – review & editing. LA-R: Conceptualization, Project administration, Supervision, Writing – original draft, Writing – review & editing.
