# Supplementary material for: Understanding dynamics and overlapping epidemiologies of HIV, HSV-2, chlamydia, gonorrhea, and syphilis in sexual networks of men who have sex with men
Source: Front Public Health. 2024 Apr 2;12:1335693. doi: 10.3389/fpubh.2024.1335693 (PMC11018893; doi:10.3389/fpubh.2024.1335693)
Supplement: Supplementary file 1 [file Data_Sheet_1.docx]

Supplementary Material

**Table of Contents**

**Table S1. Model parameters**2

**Figure S1. Scatterplots depicting the relationship between the prevalences of ever infection for the five STIs across the 500 simulated STI epidemics in the 500 diverse MSM sexual networks**4

**Table S2. Correlations between ever infection prevalences for each pair of STIs, along with their 95% confidence intervals, estimated using A) Spearman's rank correlation coefficient and B) the maximal information coefficient. The correlation coefficients were computed across the 500 simulated STI epidemics in the 500 diverse MSM sexual networks**5

**Figure S2. Scatterplots depicting the relationship between the incidence rates of infection for the five STIs across the 500 simulated STI epidemics in the 500 diverse MSM sexual networks**6

**Table S3. Correlations between incidence rates of infection for each pair of STIs, along with their 95% confidence intervals, estimated using A) Spearman's rank correlation coefficient and B) the maximal information coefficient. The correlation coefficients were computed across the 500 simulated STI epidemics in the 500 diverse MSM sexual networks7**

**Table S4. Distribution of mean proportion of the population with no, one, two, three, four, and five specific concurrent STI infections across the 500 simulated STI epidemics in the 500 diverse MSM sexual networks. Infection presence is denoted by '1' and absence by '0'** 8

**Table S5. Distribution of mean proportion of the population with no, one, two, three, four, and five specific ever STI infections across the 500 simulated STI epidemics in the 500 diverse MSM sexual networks. Infection presence is denoted by '1' and absence by '0'**9

**References**10

# Table S1. Model parameters

| **Parameters** | **Value** | **Sources** |
| --- | --- | --- |
| **HIV** | | |
| HIV transmission probability per vaginal sex act |  |  |
| Acute infection | 0.0360 | Published literature (1) |
| Chronic infection | 0.0008 | Published literature (2, 3) |
| Advanced infection | 0.0042 | Published literature (2, 3) |
| HIV cofactor for anal sex transmission^*^ | 1.5 | Published literature (4, 5) |
| Duration of each HIV stage |  |  |
| From acute to chronic | 49 days | Published literature (2, 3, 6) |
| From chronic to AIDS | 9.0 years | Published literature (3, 7, 8) |
| From AIDS to death | 2.0 years | Published literature (2, 3) |
| **HSV-2** |  |  |
| HSV-2 transmission probability per anal sex act |  |  |
| Primary infection | 0.004 | Published literature (9) |
| Latent infection | 0.0 | Published literature (9) |
| Reactivation | 0.004 | Published literature (9) |
| Duration of each HSV-2 stage |  |  |
| From primary to latent | 20 days | Published literature (9) |
| From latent to reactivation | 78.5 days | Published literature (9) |
| From reactivation to latent | 12.8 days | Published literature (9) |
| HSV-2 shedding frequency | 14% | Published literature (9, 10) |
| **Chlamydia** |  |  |
| Chlamydia transmission probability per anal sex act | 0.17 | Published literature (11, 12) |
| Proportion of chlamydia infections |  |  |
| Becoming symptomatic among males | 0.30 | Published literature (13) |
| Successfully treated | 0.70 | Published literature (13) |
| Immune after treatment | 0.5 | Published literature (13) |
| Duration of |  |  |
| Symptomatic chlamydia infection | 16 weeks | Published literature (13) |
| Asymptomatic chlamydia infection | 90 weeks | Published literature (13) |
| Immunity | 520 weeks | Published literature (13) |
| **Gonorrhea** |  |  |
| Gonorrhea transmission probability per anal sex act | 0.46 | Published literature (14) |
| Proportion of gonorrhea infections |  |  |
| Becoming symptomatic among males | 0.64 | Published literature (13, 15, 16) |
| Successfully treated | 0.70 | Published literature (13) |
| Immune after treatment | 0.5 | Published literature (13) |
| Duration of |  |  |
| Symptomatic or asymptomatic infection if untreated | 20 weeks | Published literature (13) |
| Immunity | 52 weeks | Published literature (13) |
| **Syphilis** |  |  |
| Syphilis transmission probability per anal sex act | 0.20 | Average of male-to-female and female-to-male transmission probability (13) |
| Duration of |  |  |
| Incubation | 4.4 weeks | Published literature (13) |
| Primary syphilis | 6.6 weeks | Published literature (13) |
| Secondary syphilis | 15.6 weeks | Published literature (13) |
| Latent syphilis | 520 weeks | Published literature (13) |
| Proportion of |  |  |
| Primary cases seronegative after successful treatment | 0.40 | Published literature (13) |
| Primary syphilis cases successfully treated | 0.70 | Published literature (13) |
| Secondary syphilis cases successfully treated^†^ | 30% lower than for primary syphilis | Published literature^†^ (13, 17) |
| Reduction in health seeking behavior for secondary syphilis compared to primary syphilis | 0.5 | Published literature (13) |
| Duration from |  |  |
| Recovery in early disease to seronegative susceptible | 26 weeks | Published literature (13) |
| Recovery in late disease to seronegative susceptible | 52 weeks | Published literature (13) |
| **Sexual behavior** |  |  |
| Long-term (spousal) partnership formation rate | 0.12 | Representative value informed by rate of spousal partnership formation rate among the heterosexual population (18, 19) |
| Mean duration |  |  |
| Long-term (spousal) sexual partnership | 5 years | Representative value informed by duration of spousal partnerships among the heterosexual population (18, 19) |
| Short-term (casual) sexual partnership | 2 weeks | Representative value and informed by previous work (20, 21) |
| Number of short-term (casual) sex partners among individuals not in long-term (spousal) partnerships over the last year |  |  |
| Mean | 0.0-5.0 | Representative range informed by analyses of empirical data (22) |
| Variance | 0.0-5.0 |  |
| Number of short-term (casual) sex partners among individuals in long-term (spousal) partnerships over the last year |  |  |
| Mean | 0.0-2.5 | Representative range informed by analyses of empirical data (22) |
| Variance | 0.0-2.5 |  |
| Tuning parameter |  |  |
| Degree correlation (*κ_corr_*) | -10.0-10.0 | Representative range to generate wide variation in degree correlation(19) |
| Clustering (*κ_clus_*) | 0.0-20.0 | Representative range to generate wide variation in clustering (19) |
| **Demographic factors** |  |  |
| Natural mortality rate per year by age group |  |  |
| 0-4 years | 0.04 |  |
| 5-69 years | 0.0026 |  |
| 70+ years | 0.0998 |  |

HIV denotes human immunodeficiency virus; HSV-2, herpes simplex virus type 2.

^*^The transmission probability per one anal sex act is determined as the product of HIV transmission probability per coital act for vaginal sex time the HIV cofactor for anal sex transmission.

^†^Probability of treatment success was assumed to be 30% lower for secondary syphilis than for primary syphilis, due to the greater difficulty of diagnosing secondary syphilis symptoms and the lower efficacy of penicillin in treating syphilis of longer duration (13, 17).

# Figure S1. Scatterplots depicting the relationship between the prevalences of ever infection for the five STIs across the 500 simulated STI epidemics in the 500 diverse MSM sexual networks.

**
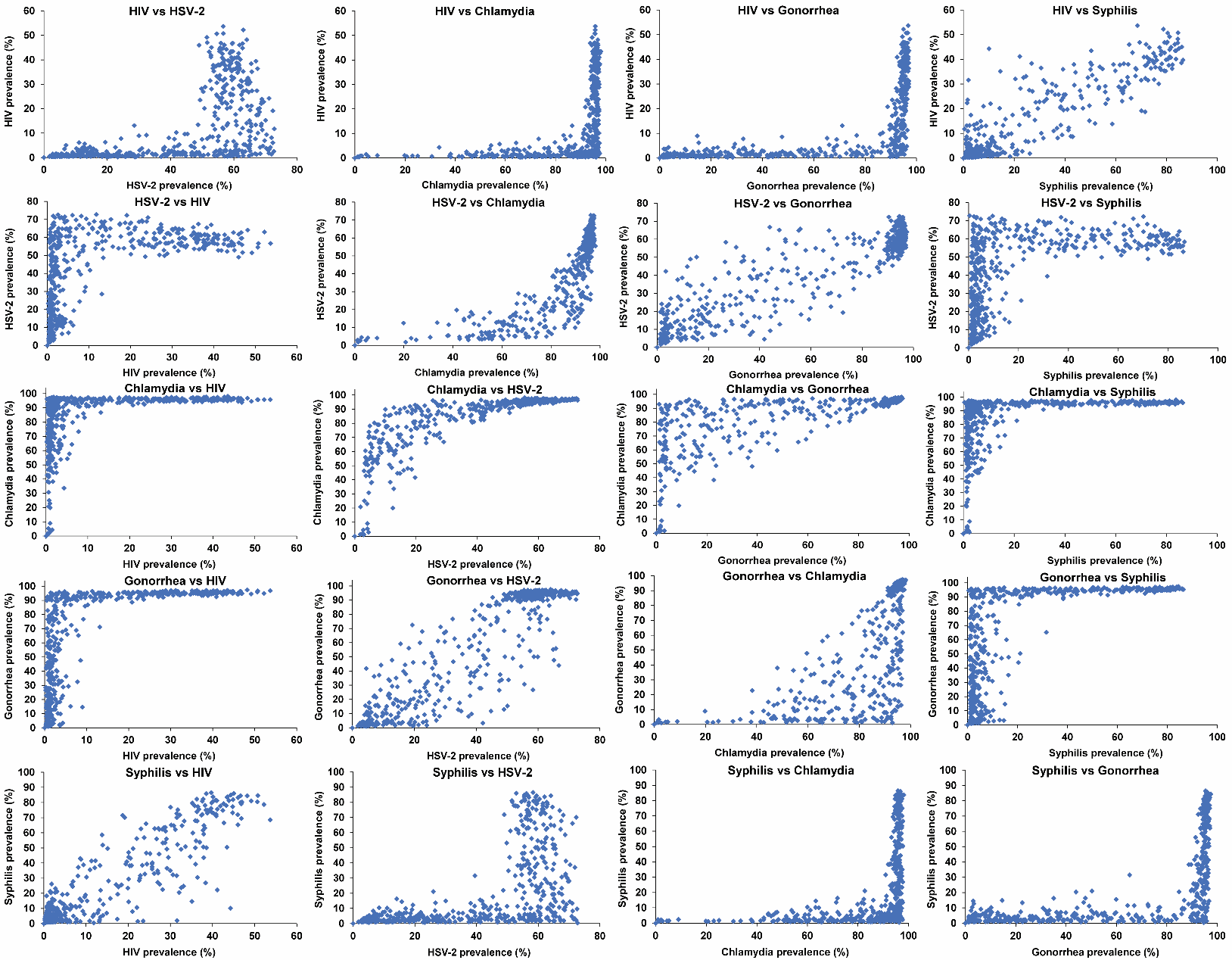
**

HIV denotes human immunodeficiency virus; HSV-2, herpes simplex virus type 2; MSM, men who have sex with men; STI, sexually transmitted infection.

**Table S2. Correlations between ever infection prevalences for each pair of STIs, along with their 95% confidence intervals, estimated using A) Spearman's rank correlation coefficient and B) the maximal information coefficient. The correlation coefficients were computed across the 500 simulated STI epidemics in the 500 diverse MSM sexual networks.**

| 1. **Spearman's rank correlation coefficient (SRCC)** | | | | | | **Average** |
| --- | --- | --- | --- | --- | --- | --- |
|  | **HIV**  **(95% CI)** | **HSV-2**  **(95% CI)** | **Chlamydia**  **(95% CI)** | **Gonorrhea**  **(95% CI)** | **Syphilis**  **(95% CI)** | **SRCC** |
| **HIV** |  | 0.66  (0.61, 0.70) | 0.66  (0.61, 0.71) | 0.85  (0.82, 0.87) | 0.78  (0.73, 0.82) | 0.74 |
| **HSV-2** | 0.66  (0.61, 0.70) |  | 0.89  (0.86, 0.91) | 0.85  (0.82, 0.87) | 0.62  (0.57, 0.68) | 0.76 |
| **Chlamydia** | 0.66  (0.61, 0.71) | 0.89  (0.86, 0.91) |  | 0.84  (0.82, 0.87) | 0.65  (0.60, 0.70) | 0.76 |
| **Gonorrhea** | 0.85  (0.82, 0.87) | 0.85  (0.82, 0.87) | 0.84  (0.82, 0.87) |  | 0.76  (0.72, 0.80) | 0.83 |
| **Syphilis** | 0.78  (0.73, 0.82) | 0.62  (0.57, 0.68) | 0.65  (0.60, 0.70) | 0.76  (0.72, 0.80) |  | 0.70 |
| 1. **Maximal information coefficient (MIC)** | | | | | | |
|  | **HIV**  **(95% CI)** | **HSV-2**  **(95% CI)** | **Chlamydia**  **(95% CI)** | **Gonorrhea**  **(95% CI)** | **Syphilis**  **(95% CI)** | **MIC** |
| **HIV** |  | 0.67  (0.58, 0.76) | 0.60  (0.53, 0.67) | 0.83  (0.76, 0.91) | 0.72  (0.66, 0.79) | 0.71 |
| **HSV-2** | 0.67  (0.58, 0.76) |  | 0.91  (0.84, 0.97) | 0.91  (0.85, 0.96) | 0.64  (0.56, 0.71) | 0.78 |
| **Chlamydia** | 0.60  (0.53, 0.67) | 0.91  (0.84, 0.97) |  | 0.82  (0.76, 0.87) | 0.60  (0.53, 0.67) | 0.73 |
| **Gonorrhea** | 0.83  (0.76, 0.91) | 0.91  (0.85, 0.96) | 0.82  (0.76, 0.87) |  | 0.73  (0.63, 0.81) | 0.82 |
| **Syphilis** | 0.72  (0.66, 0.79) | 0.64  (0.56, 0.71) | 0.60  (0.53, 0.67) | 0.73  (0.63, 0.81) |  | 0.67 |

CI denotes confidence interval; HIV, human immunodeficiency virus; HSV-2, herpes simplex virus type 2; MSM, men who have sex with men; STI, sexually transmitted infection.

**Figure S2. Scatterplots depicting the relationship between the incidence rates of infection for the five STIs across the 500 simulated STI epidemics in the 500 diverse MSM sexual networks.**

**
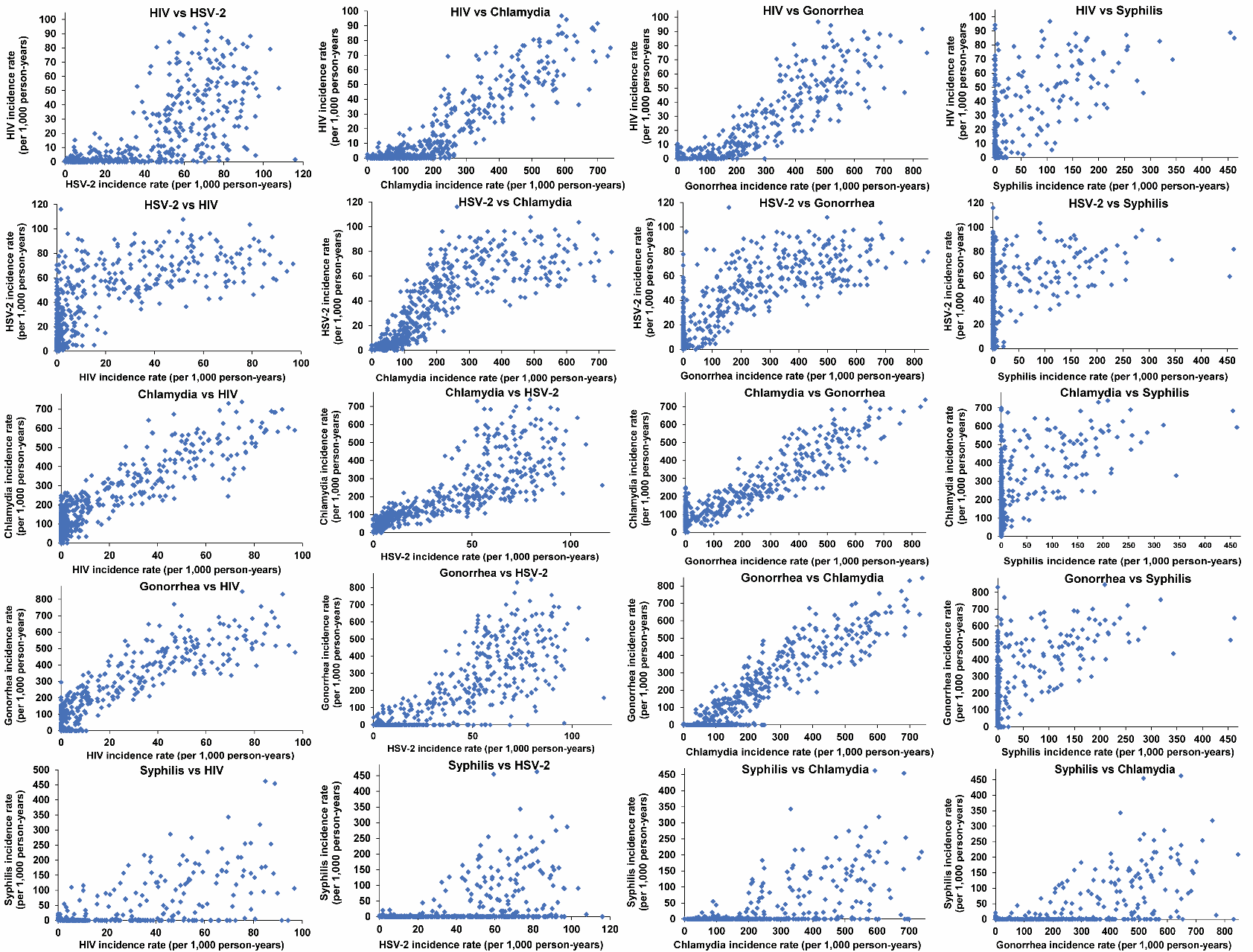
**

HIV denotes human immunodeficiency virus; HSV-2, herpes simplex virus type 2; MSM, men who have sex with men; STI, sexually transmitted infection.

**Table S3. Correlations between incidence rates of infection for each pair of STIs, along with their 95% confidence intervals, estimated using A) Spearman's rank correlation coefficient and B) the maximal information coefficient. The correlation coefficients were computed across the 500 simulated STI epidemics in the 500 diverse MSM sexual networks.**

| 1. **Spearman's rank correlation coefficient (SRCC)** | | | | | | **Average** |
| --- | --- | --- | --- | --- | --- | --- |
|  | **HIV**  **(95% CI)** | **HSV-2**  **(95% CI)** | **Chlamydia**  **(95% CI)** | **Gonorrhea**  **(95% CI)** | **Syphilis**  **(95% CI)** | **SRCC** |
| **HIV** |  | 0.75  (0.70, 0.78) | 0.81  (0.77, 0.85) | 0.83  (0.79, 0.87) | 0.50  (0.42, 0.57) | 0.72 |
| **HSV-2** | 0.75  (0.70, 0.78) |  | 0.90  (0.88, 0.91) | 0.81  (0.78, 0.84) | 0.42  (0.34, 0.49) | 0.72 |
| **Chlamydia** | 0.81  (0.77, 0.85) | 0.90  (0.88, 0.91) |  | 0.87  (0.84, 0.89) | 0.50  (0.43, 0.57) | 0.77 |
| **Gonorrhea** | 0.83  (0.79, 0.87) | 0.81  (0.78, 0.84) | 0.87  (0.84, 0.89) |  | 0.50  (0.42, 0.58) | 0.75 |
| **Syphilis** | 0.50  (0.42, 0.57) | 0.42  (0.34, 0.49) | 0.50  (0.43, 0.57) | 0.50  (0.42, 0.58) |  | 0.48 |
| 1. **Maximal information coefficient (MIC)** | | | | | | |
|  | **HIV**  **(95% CI)** | **HSV-2**  **(95% CI)** | **Chlamydia**  **(95% CI)** | **Gonorrhea**  **(95% CI)** | **Syphilis**  **(95% CI)** | **MIC** |
| **HIV** |  | 0.69  (0.62, 0.77) | 0.76  (0.70, 0.83) | 0.80  (0.75, 0.86) | 0.37  (0.32, 0.43) | 0.66 |
| **HSV-2** | 0.69  (0.62, 0.77) |  | 0.91  (0.86, 0.95) | 0.83  (0.76, 0.90) | 0.35  (0.31, 0.39) | 0.70 |
| **Chlamydia** | 0.76  (0.70, 0.83) | 0.91  (0.86, 0.95) |  | 0.85  (0.77, 0.91) | 0.40  (0.35, 0.46) | 0.73 |
| **Gonorrhea** | 0.80  (0.75, 0.86) | 0.83  (0.76, 0.90) | 0.85  (0.77, 0.91) |  | 0.38  (0.34, 0.44) | 0.72 |
| **Syphilis** | 0.37  (0.32, 0.43) | 0.35  (0.31, 0.39) | 0.40  (0.35, 0.46) | 0.38  (0.34, 0.44) |  | 0.38 |

CI denotes confidence interval; HIV, human immunodeficiency virus; HSV-2, herpes simplex virus type 2; MSM, men who have sex with men; STI, sexually transmitted infection.

**Table S4. Distribution of mean proportion of the population with no, one, two, three, four, and five specific concurrent STI infections across the 500 simulated STI epidemics in the 500 diverse MSM sexual networks. Infection presence is denoted by '1' and absence by '0'.**

| **HIV** | **HSV-2** | **Chlamydia** | **Gonorrhea** | **Syphilis** | **Proportion (in %)** |
| --- | --- | --- | --- | --- | --- |
| 0 | 0 | 0 | 0 | 0 | 48.56 |
| 0 | 0 | 0 | 0 | 1 | 0.90 |
| 0 | 0 | 0 | 1 | 0 | 0.75 |
| 0 | 0 | 1 | 0 | 0 | 4.03 |
| 0 | 1 | 0 | 0 | 0 | 29.11 |
| 1 | 0 | 0 | 0 | 0 | 2.91 |
| 0 | 0 | 1 | 0 | 1 | 0.16 |
| 0 | 0 | 0 | 1 | 1 | 0.07 |
| 0 | 0 | 1 | 1 | 0 | 0.21 |
| 0 | 1 | 0 | 0 | 1 | 1.27 |
| 0 | 1 | 1 | 0 | 0 | 2.05 |
| 0 | 1 | 0 | 1 | 0 | 0.69 |
| 1 | 0 | 0 | 0 | 1 | 0.47 |
| 1 | 0 | 1 | 0 | 0 | 0.40 |
| 1 | 0 | 0 | 1 | 0 | 0.21 |
| 1 | 1 | 0 | 0 | 0 | 5.57 |
| 0 | 0 | 1 | 1 | 1 | 0.02 |
| 0 | 1 | 1 | 0 | 1 | 0.14 |
| 0 | 1 | 0 | 1 | 1 | 0.09 |
| 0 | 1 | 1 | 1 | 0 | 0.10 |
| 1 | 0 | 1 | 0 | 1 | 0.06 |
| 1 | 0 | 0 | 1 | 1 | 0.05 |
| 1 | 0 | 1 | 1 | 0 | 0.04 |
| 1 | 1 | 0 | 0 | 1 | 1.01 |
| 1 | 1 | 1 | 0 | 0 | 0.54 |
| 1 | 1 | 0 | 1 | 0 | 0.32 |
| 0 | 1 | 1 | 1 | 1 | 0.01 |
| 1 | 0 | 1 | 1 | 1 | 0.01 |
| 1 | 1 | 1 | 0 | 1 | 0.11 |
| 1 | 1 | 0 | 1 | 1 | 0.09 |
| 1 | 1 | 1 | 1 | 0 | 0.05 |
| 1 | 1 | 1 | 1 | 1 | 0.01 |

HIV denotes human immunodeficiency virus; HSV-2, herpes simplex virus type 2; MSM, men who have sex with men; STI, sexually transmitted infection.

**Table S5. Distribution of mean proportion of the population with no, one, two, three, four, and five specific ever STI infections across the 500 simulated STI epidemics in the 500 diverse MSM sexual networks. Infection presence is denoted by '1' and absence by '0'.**

| **HIV** | **HSV-2** | **Chlamydia** | **Gonorrhea** | **Syphilis** | **Proportion (in %)** |
| --- | --- | --- | --- | --- | --- |
| 0 | 0 | 0 | 0 | 0 | 13.64 |
| 0 | 0 | 0 | 0 | 1 | 0.18 |
| 0 | 0 | 0 | 1 | 0 | 1.04 |
| 0 | 0 | 1 | 0 | 0 | 19.09 |
| 0 | 1 | 0 | 0 | 0 | 0.64 |
| 1 | 0 | 0 | 0 | 0 | 0.09 |
| 0 | 0 | 1 | 0 | 1 | 0.76 |
| 0 | 0 | 0 | 1 | 1 | 0.06 |
| 0 | 0 | 1 | 1 | 0 | 15.68 |
| 0 | 1 | 0 | 0 | 1 | 0.01 |
| 0 | 1 | 1 | 0 | 0 | 5.79 |
| 0 | 1 | 0 | 1 | 0 | 0.18 |
| 1 | 0 | 0 | 0 | 1 | 0.00 |
| 1 | 0 | 1 | 0 | 0 | 0.34 |
| 1 | 0 | 0 | 1 | 0 | 0.04 |
| 1 | 1 | 0 | 0 | 0 | 0.01 |
| 0 | 0 | 1 | 1 | 1 | 4.27 |
| 0 | 1 | 1 | 0 | 1 | 0.35 |
| 0 | 1 | 0 | 1 | 1 | 0.01 |
| 0 | 1 | 1 | 1 | 0 | 17.73 |
| 1 | 0 | 1 | 0 | 1 | 0.03 |
| 1 | 0 | 0 | 1 | 1 | 0.01 |
| 1 | 0 | 1 | 1 | 0 | 1.73 |
| 1 | 1 | 0 | 0 | 1 | 0.00 |
| 1 | 1 | 1 | 0 | 0 | 0.14 |
| 1 | 1 | 0 | 1 | 0 | 0.01 |
| 0 | 1 | 1 | 1 | 1 | 8.73 |
| 1 | 0 | 1 | 1 | 1 | 1.91 |
| 1 | 1 | 1 | 0 | 1 | 0.01 |
| 1 | 1 | 0 | 1 | 1 | 0.00 |
| 1 | 1 | 1 | 1 | 0 | 2.70 |
| 1 | 1 | 1 | 1 | 1 | 4.82 |

HIV denotes human immunodeficiency virus; HSV-2, herpes simplex virus type 2; MSM, men who have sex with men; STI, sexually transmitted infection.

**References**

1. Wawer MJ, Gray RH, Sewankambo NK, Serwadda D, Li X, Laeyendecker O, Kiwanuka N, Kigozi G, Kiddugavu M, Lutalo T, Nalugoda F, Wabwire-Mangen F, Meehan MP, Quinn TC. Rates of HIV-1 transmission per coital act, by stage of HIV-1 infection, in Rakai, Uganda. J Infect Dis. 2005 May 1;191(9):1403-9. Cited in: Pubmed; PMID 15809897.

2. Pinkerton SD. Probability of HIV transmission during acute infection in Rakai, Uganda. AIDS Behav. 2008 Sep;12(5):677-84. eng. Epub 2007/12/08. doi:10.1007/s10461-007-9329-1. Cited in: Pubmed; PMID 18064559.

3. Hollingsworth TD, Anderson RM, Fraser C. HIV-1 transmission, by stage of infection. J Infect Dis. 2008 Sep 1;198(5):687-93. eng. Epub 2008/07/30. doi:10.1086/590501. Cited in: Pubmed; PMID 18662132.

4. Abu-Raddad LJ, Longini IM, Jr. No HIV stage is dominant in driving the HIV epidemic in sub-Saharan Africa. AIDS. 2008 May 31;22(9):1055-61. Epub 2008/06/04. doi:10.1097/QAD.0b013e3282f8af84. Cited in: Pubmed; PMID 18520349.

5. Baggaley RF, White RG, Boily MC. HIV transmission risk through anal intercourse: systematic review, meta-analysis and implications for HIV prevention. Int J Epidemiol. 2010 Aug;39(4):1048-63. Epub 2010/04/22. doi:10.1093/ije/dyq057. Cited in: Pubmed; PMID 20406794.

6. Patel P, Borkowf CB, Brooks JT, Lasry A, Lansky A, Mermin J. Estimating per-act HIV transmission risk: a systematic review. AIDS. 2014 Jun 19;28(10):1509-19. Epub 2014/05/09. doi:10.1097/QAD.0000000000000298. Cited in: Pubmed; PMID 24809629.

7. Morgan D, Whitworth J. The natural history of HIV-1 infection in Africa. Nat Med. 2001 Feb;7(2):143-5. Cited in: Pubmed; PMID 11175832.

8. UNAIDS. UNAIDS Reference Group on Estimates, Modelling and Projections. 2007.

9. Abu-Raddad LJ, Magaret AS, Celum C, Wald A, Longini IM, Jr., Self SG, Corey L. Genital herpes has played a more important role than any other sexually transmitted infection in driving HIV prevalence in Africa. PLoS One. 2008 May 21;3(5):e2230. Epub 2008/05/22. doi:10.1371/journal.pone.0002230. Cited in: Pubmed; PMID 18493617.

10. Mark KE, Wald A, Magaret AS, Selke S, Olin L, Huang ML, Corey L. Rapidly cleared episodes of herpes simplex virus reactivation in immunocompetent adults. J Infect Dis. 2008 Oct 15;198(8):1141-9. eng. Epub 2008/09/12. doi:10.1086/591913. Cited in: Pubmed; PMID 18783315.

11. Xiridou M, Vriend HJ, Lugner AK, Wallinga J, Fennema JS, Prins JM, Geerlings SE, Rijnders BJ, Prins M, de Vries HJ, Postma MJ, van Veen MG, Schim van der Loeff MF, van der Sande MA. Modelling the impact of chlamydia screening on the transmission of HIV among men who have sex with men. BMC Infect Dis. 2013 Sep 18;13:436. Epub 2013/09/21. doi:10.1186/1471-2334-13-436. Cited in: Pubmed; PMID 24047261.

12. Jenness SM, Weiss KM, Goodreau SM, Gift T, Chesson H, Hoover KW, Smith DK, Liu AY, Sullivan PS, Rosenberg ES. Incidence of Gonorrhea and Chlamydia Following Human Immunodeficiency Virus Preexposure Prophylaxis Among Men Who Have Sex With Men: A Modeling Study. Clin Infect Dis. 2017 Sep 1;65(5):712-718. Epub 2017/05/16. doi:10.1093/cid/cix439. Cited in: Pubmed; PMID 28505240.

13. Johnson LF, Geffen N. A Comparison of Two Mathematical Modeling Frameworks for Evaluating Sexually Transmitted Infection Epidemiology. Sex Transm Dis. 2016 Mar;43(3):139-46. doi:10.1097/OLQ.0000000000000412. Cited in: Pubmed; PMID 26859800.

14. Zhang L, Regan DG, Chow EPF, Gambhir M, Cornelisse V, Grulich A, Ong J, Lewis DA, Hocking J, Fairley CK. Neisseria gonorrhoeae Transmission Among Men Who Have Sex With Men: An Anatomical Site-Specific Mathematical Model Evaluating the Potential Preventive Impact of Mouthwash. Sex Transm Dis. 2017 Oct;44(10):586-592. Epub 2017/09/07. doi:10.1097/OLQ.0000000000000661. Cited in: Pubmed; PMID 28876289.

15. Rowley J, Vander Hoorn S, Korenromp E, Low N, Unemo M, Abu-Raddad LJ, Chico RM, Smolak A, Newman L, Gottlieb S. Chlamydia, gonorrhoea, trichomoniasis and syphilis: global prevalence and incidence estimates, 2016. Bulletin of the World Health Organization. 2019;97(8):548.

16. Newman L, Rowley J, Vander Hoorn S, Wijesooriya NS, Unemo M, Low N, Stevens G, Gottlieb S, Kiarie J, Temmerman M. Global estimates of the prevalence and incidence of four curable sexually transmitted infections in 2012 based on systematic review and global reporting. PloS one. 2015;10(12):e0143304.

17. Ganesan A, Mesner O, Okulicz JF, O'Bryan T, Deiss RG, Lalani T, Whitman TJ, Weintrob AC, Macalino G, Agan BK, Infectious Disease Clinical Research Program HIVSTIWG. A single dose of benzathine penicillin G is as effective as multiple doses of benzathine penicillin G for the treatment of HIV-infected persons with early syphilis. Clin Infect Dis. 2015 Feb 15;60(4):653-60. Epub 2014/11/13. doi:10.1093/cid/ciu888. Cited in: Pubmed; PMID 25389249.

18. MEASURE DHS. Demographic and health surveys. Calverton: ICF Macro; May 19. Available from: <http://www.measuredhs.com/>.

19. Omori R, Abu-Raddad LJ. Sexual network drivers of HIV and herpes simplex virus type 2 transmission. AIDS. 2017 Jul 31;31(12):1721-1732. Epub 2017/05/18. doi:10.1097/QAD.0000000000001542. Cited in: Pubmed; PMID 28514276.

20. Abu-Raddad LJ, Longini Jr IM. No HIV stage is dominant in driving the HIV epidemic in sub-Saharan Africa. Aids. 2008;22(9):1055-1061.

21. Awad SF, Abu-Raddad LJ. Could there have been substantial declines in sexual risk behavior across sub-Saharan Africa in the mid-1990s? [Peer-reviewed study]. Epidemics. 2014 9//;8(0):9-17. doi:<http://dx.doi.org/10.1016/j.epidem.2014.06.001>.

22. Omori R, Chemaitelly H, Abu-Raddad LJ. Dynamics of non-cohabiting sex partnering in sub-Saharan Africa: a modelling study with implications for HIV transmission. Sex Transm Infect. 2015 Sep;91(6):451-7. Epub 2015/03/10. doi:10.1136/sextrans-2014-051925. Cited in: Pubmed; PMID 25746040.
